# A PRECEDE-PROCEED-based educational program: enhancing self-care and quality of life with implications for biochemical outcomes in hemodialysis patients

**DOI:** 10.3389/fpubh.2026.1766801

**Published:** 2026-03-25

**Authors:** Elly Lilianty Sjattar, Andina Setyawati, Aulia Insani Latif, Nuurhidayat Jafar, Yuliana Syam, Ummi Pratiwi Rimayanti, Gerhard Fortwengel, Nor Aziyan Binti Yahaya, Dnyanesh Limaye, Nita Hardianty

**Affiliations:** 1 Department of Medical and Surgical Nursing, Faculty of Nursing, Hasanuddin University, Makassar, South Sulawesi, Indonesia; 2 Department of Community Health Nursing, Faculty of Nursing, Hasanuddin University, Makassar, South Sulawesi, Indonesia; 3 Department of Basic Nursing, Faculty of Nursing, Hasanuddin University, Makassar, South Sulawesi, Indonesia; 4 Department of Clinical Trial, Hochschule Hannover, Hanover, Germany; 5 Department of Nursing Science, Faculty of Medicine, University of Malaya, Kuala Lumpur, Malaysia; 6 Department Research and Development, Center for Advancement in Health Sciences, Pune, India; 7 Department of Nursing, Dr. Sulaiman Al Habib Medical Hospital Riyadh, Riyadh, Saudi Arabia

**Keywords:** chronic kidney disease, health education, hemodialysis, quality of life, self-care

## Abstract

**Objective:**

This study evaluated the effectiveness of a health education program based on the PRECEDE-PROCEED model in improving self-care, QOL, and biochemical outcomes among HD patients in Indonesia.

**Methods:**

A quasi-experimental study was conducted involving 90 HD patients allocated to intervention (*n* = 45) and control (*n* = 45) groups using a systematic assignment approach. The intervention group received an eight-session PRECEDE-PROCEED-based education program over 4 weeks, while the control group received standard care. Self-care was measured using the Chronic Kidney Disease Self-Management (CKDSM) Questionnaire, QOL was assessed using the SF-36, and biochemical outcomes were evaluated via serum urea and creatinine levels. Data were analyzed using appropriate parametric and non-parametric statistical tests, and effect sizes were calculated using Cohen’s *d*.

**Results:**

Post-intervention, the intervention group showed significantly greater improvements in self-care (mean difference = 18.33; *p* < 0.001), QOL (mean difference = 17.25; *p* < 0.001), and reductions in urea and creatinine levels (*p* < 0.001 and *p* = 0.006, respectively), compared to the control group. Large effect sizes were observed for self-care and QOL.

**Conclusion:**

The PRECEDE-PROCEED–based educational intervention was associated with improvements in self-care and quality of life, and with favorable short-term changes in selected clinical outcomes among patients undergoing hemodialysis.

## 1 Introduction

Chronic Kidney Disease is affecting more people around the world every year, leading to increased incidences of End-Stage Renal Disease (ESRD) that require hemodialysis (HD) as a life-sustaining therapy (1). Indonesia, nation with the fourth-highest population count, has experienced a significant growth in the HD patient population. According to the Indonesian Renal Registry (IRR), new HD patients grew from 15,128 in 2013 to 61,786 in 2020. Between 2013 and 2020, the number of active HD patients rose from 21,759 to 130,931. Given the estimated 270 million people in the world, this rise translates into an incidence rate of 251 per million and a prevalence rate of 499 per million. In Indonesia, HD is used for almost 98% of ESRD patients, with a reported 53.7% 60-month survival rate (2).

Given the increasing number of HD patients, promoting adequate self-care has become crucial aspect of patient management. The ability to perform adequate self-care is fundamental for maintaining the health and well-being of patients undergoing HD (3). While hemodialysis is vital for sustaining life in ESRD patients, it presents long-term challenges to maintaining adequate self-care and, consequently, affects their overall Quality of life (QOL) (4). Patients undergoing HD must manage fluid and dietary restrictions, adhere to strict treatment regimens, and must also cope with declines in physical and psychosocial functioning. Studies have reported that Indonesian patients undergoing HD often demonstrate low to moderate levels of engagement in these self-care activities, resulting in lower QOL (5, 6). Moreover, the burden of care often extends to family members with moderate to severe impacts on their financial stability, family and social life, autonomy, and emotional well-being (7).

Beyond its impact on QOL, inadequate self-care among HD patients also contributes to poor clinical outcomes, particularly affecting biochemical parameters such as urea and creatinine levels (8, 9). A major clinical concern in HD patients is the persistent elevation of urea and creatinine levels despite regular dialysis (10). Although HD partially mitigates these imbalances, improper self-care behaviors can exacerbate the condition, leading to worse clinical trajectories. Accordingly, interventions that address behavioral, environmental, and psychosocial factors are imperative to improve both clinical parameters and QOL outcome in this vulnerable population (11).

Educational interventions have been shown to foster better self-care behaviors among HD patients, leading to enhance both clinical outcomes and QOL in patients undergoing HD (12). Health education not only increases patient knowledge but also strengthens self-management skills capabilities and sustained adherence to treatment regimens (13, 14). Health promotion models provide a methodical systematic approach to the planning, implementation, and evaluation of such educational programs (15). However, despite accumulating evidence of their efficacy, a systematically structured educational program based on health promotion models is not yet widely implemented in Indonesian HD care settings.

The PRECEDE-PROCEED model, developed by Green and Kreuter (16), provides a robust framework for conducting needs assessment, designing, implementing, and evaluating health promotion education programs. The model is grounded in the concept that effective health education should be precede by thorough assessment process (PRECEDE: Predisposing, Reinforcing, and Enabling Constructs in Educational Diagnosis and Evaluation) and should process with proper policy, environmental, and regulatory considerations followed by implementation and evaluation (PROCEED: Policy, Regulatory, and Organizational Constructs in Educational and Environmental Development). This model allows the integration of educational, ecological, and policy-level determinants to develop interventions that are tailored to the specific needs.

Numerous studies have demonstrated how successful it is to use this model when creating educational initiatives to encourage individuals with chronic illnesses to make changes to their self-care practices (17). In the scope of HD care, the PRECEDE-PROCEED model provides a promising approach for systematically identifying factors influencing patient self-care and guiding the development of targeted educational programs to improve self-care and QOL.

Thus, this study aimed to develop and evaluate a PRECEDE-PROCEED-based educational program to enhance self-care behaviors and improve QOL among Indonesian patients undergoing HD. Additionally, the study sought to explore potential effect on key biochemical parameters, specifically urea and creatinine levels.

## 2 Methods

### 2.1 Study design

This study employed a quasi-experimental design with parallel intervention and control groups. Group allocation was conducted using a systematic assignment approach based on enrolment order, in which participants with odd enrolment numbers were assigned to the intervention group, while those with even numbers were assigned to the control group.

### 2.2 Participant recruitment

Participants were allocated to either the intervention or control group at a 1:1 ratio. The required sample size was calculated using the two-sample proportion formula (18). The formula used was:


$$n=\frac{2{o^{\prime }}^{2}{\left(Z_{1-\alpha /2}+Z_{1-\beta }\right)}^{2}}{{\left(\mu_{1}-\mu_{2}\right)}^{2}}$$

$$n=\frac{2{\left(\frac{3,11}{2}\right)}^{2}{\left(1,96+0,842\right)}^{2}}{{\left(7,7-6,7\right)}^{2.}}$$

$$n=\frac{4,83605\;X\;7,851204}{1}$$

n=38each group

After adjusting for a 10% potential attrition rate, the final sample size was 84 participants, with 42 assigned to the intervention group and 42 to the control group. The sample size calculation was explicitly linked to the primary outcomes of the study, with self-care behavior serving as the main reference outcome for estimation, as it represents the principal behavioral target of the PRECEDE–PROCEED–based educational intervention.

The inclusion criteria were: patients diagnosed with Stage 4–5 CKD, undergoing HD for at least 3 months, receiving HD twice a week on a regular basis, aged ≥18 years, physically capable of performing routine daily activities, and demonstrating insufficient knowledge. Participants were recruited through probability sampling using a simple random technique.

### 2.3 Intervention

Both groups received a printed educational booklet. The control group received standard health education according to hospital protocol. The intervention group, in addition, participated in a structured educational program based on the PRECEDE-PROCEED model. The program included eight sessions, conducted twice weekly, each lasting 10–15 min and covering distinct topics related to self-care, reinforced by follow-up activities.

The PRECEDE-PROCEED framework guided the planning and implementation of the program. In the PRECEDE phase, an assessment of patients’ needs was conducted by identifying predisposing, reinforcing, and enabling factors influencing self-care behaviors. Predisposing factors included patients’ knowledge, attitudes, and beliefs about self-care; reinforcing factors involved encouragement from family members and healthcare providers; and enabling factors included the availability of booklets, nurse support, and time during hemodialysis sessions to participate in education. This assessment informed the content of the educational sessions to ensure relevance and patient-centeredness.

In the PROCEED phase, the intervention was implemented through structured sessions addressing fluid and dietary management, medication adherence, vascular access care, infection prevention, physical activity, and coping strategies. Each session emphasized practical skills, patient participation, and follow-up reinforcement. The outcomes were evaluated not only in terms of knowledge and self-care behavior but also through quality of life measures and biochemical markers (serum urea and creatinine). This structured approach ensured that the program was comprehensive, evidence-based, and capable of addressing the multidimensional challenges faced by hemodialysis patients.

### 2.4 Data collection

Data collection occurred at two time points: pre-intervention (baseline) and after completion of the intervention program (post-intervention). To evaluate self-care behaviors, the Chronic Kidney Disease Self-Management (CKDSM) Questionnaire was administered. This instrument, adapted from Almutary and Tayyib (19) and Smekal et al. (20) consists of 25 items grouped into three subscales: self-care maintenance, self-care management, and self-care confidence. Each item is rated on a 4-point Likert scale ranging from 1 (not confident) to 4 (very confident), producing a total score between 25 and 100, with higher scores reflecting better self-care ability. The questionnaire demonstrated excellent reliability, with Cronbach’s alpha of 0.921 during the pilot test and 0.912 in the main study. Health-related quality of life was assessed using the Kidney Disease Quality of Life 36-Item Short Form (KDQOL-36™), a validated instrument that includes the Physical Component Summary, Mental Component Summary, Symptoms and Problems, Burden of Kidney Disease, and Effects of Kidney Disease subscales (21). The Indonesian version used in this study, translated and validated by Supriyadi et al. (22), demonstrated adequate internal consistency with a Cronbach’s alpha of 0.798. In addition, biochemical outcomes were measured through laboratory examinations of serum urea and creatinine levels. Blood samples were collected by trained healthcare professionals following standardized procedures and analyzed at the Makassar Public Health Laboratory Center.

In addition, a standardized laboratory form was used to record the serum urea and creatinine levels. Blood samples were collected by trained healthcare professionals and analyzed at the Makassar Public Health Laboratory Center.

### 2.5 Study outcomes

The primary outcomes of this study were self-care behavior and self-care–related knowledge, reflecting the main behavioral and cognitive objectives of the PRECEDE–PROCEED–based educational intervention.

Secondary outcomes included health-related quality of life and biochemical parameters, specifically serum urea and creatinine levels, which were evaluated to explore broader patient-centered and clinical changes associated with improvements in self-care and knowledge.

### 2.6 Data analysis

Data analysis was conducted based on the distributional characteristics of the variables. Normality was assessed prior to analysis to determine the appropriate statistical approach. For variables with normal distribution, parametric tests were applied, whereas non-parametric tests were used for variables that did not meet normality assumptions.

Within-group comparisons (pre–post intervention) were performed using paired *t*-tests or Wilcoxon signed-rank tests, while between-group comparisons were conducted using independent *t*-tests or Mann–Whitney U tests, as appropriate. These analyses addressed distinct analytical objectives and were therefore considered complementary rather than redundant. Effect sizes were calculated using Cohen’s d to provide an estimate of the magnitude of observed changes beyond statistical significance.

Given the exploratory nature of this study and the predefined primary outcomes, formal adjustment for multiple comparisons was not applied. Consequently, the results should be interpreted with caution, as the use of multiple statistical tests may increase the risk of type I error.

### 2.7 Ethical approval

This study received ethical approval from the Institutional Ethics Committee of the Faculty of Nursing, Hasanuddin University, Makassar, Indonesia (Approval No. 158/UN4.18.3/TP.01.02/2025). The study was conducted in accordance with the principles of the Declaration of Helsinki. Written informed consent was obtained from all participants prior to enrolment, and participants were assured of the confidentiality and anonymity of their data throughout the study.

## 3 Results

The effectiveness of the PRECEDE-PROCEED-based educational program was evaluated through changes in self-care behaviors, quality of life (QOL), and biochemical outcomes. The outcomes were assessed by comparing pre- and post-intervention measures within and between the intervention and control groups.

Baseline demographic and clinical characteristics were first analyzed to ensure the comparability between groups. Subsequent analyses focused on evaluating changes in self-care, QOL, and serum urea and creatinine levels following the intervention. The results are summarized in Tables 1, 2.

**Table 1:** Baseline demographic and clinical characteristics of respondents in the intervention and control groups (*n* = 90).

Variables	Intervention group (*n* = 45)	Control group (*n* = 45)	*p*-value
Mean (±SD)			
Age (years)	48.87 ± 11.240	46.96 ± 11.935	0.436^a^
Duration of hemodialysis (months)	12.8 ± 14.175	16.51 ± 13.024	0.199^a^
Body weight (kg)	57.67 ± 9.501	58.58 ± 9.799	0.639^a^
n (%)			
Gender			
Male	27 (30.0%)	28 (31.1%)	0.829^b^
Female	18 (20.0%)	17 (18.9%)	
Marital status			
Not married	4 (4.4%)	2 (2.2%)	0.574^b^
Married	39 (43.3%)	42 (46.7%)	
Widow/widower	2 (2.2%)	1 (1.1%)	
Education level			
Elementary school	2 (2.2%)	8 (8.9%)	0.106^b^
Junior high school	6 (6.7%)	9 (10.0%)	
Senior high school	20 (22.2%)	18 (20.0%)	
Bachelor’s degree	17 (18.9%)	10 (11.1%)	
Occupation			
Housewife/unemployed	7 (7.8%)	15 (16.7%)	0.111^b^
Self-employed	10 (11.1%)	12 (13.3%)	
Private employee	13 (14.4%)	6 (6.7%)	
Civil servant	15 (16.7%)	12 (13.3%)	
Comorbidities			
No comorbidity	5 (5.6%)	7 (7.8%)	0.183^b^
Hypertension	20 (22.2%)	18 (20.0%)	
Diabetes mellitus	11 (12.2%)	17 (18.9%)	
Gout arthritis	5 (5.6%)	3 (3.3%)	
Heart disease	4 (4.4%)	0 (0.0%)	

^a^Independent *t*-test, ^b^Chi-square test.

**Table 2:** Comparison of self-care, quality of life, urea, and creatinine levels between the intervention and control groups before and after the intervention (*n* = 90).

Variable	Intervention group			Pair difference Mean	*P*-value	Control group		Pair difference Mean	Mean difference (MD)	*P*-value	
	Time (Pre-test/Post-test)	Mean (SD)	Median (min-max)			Mean (SD)	Median (min-max)				
Self-care	Pre-test	63.09 (12.93)	60.00 (44–92)	18.33	0.001^a^	67.64 (15.37)	72.00 (44–92)	2.47	−4.56	0.003^a^	0.177^c^
	Post-test	81.42 (9.67)	84.00 (50–93)			70.11 (14.16)	73.00 (46–92)		11.31		0.001^c^
Quality of life	Pre-test	33.85 (7.49)	32.91 (24–54)	17.25	0.001^a^	38.33 (7.86)	35.62 (24–55)	2.73	−4.48	0.001^a^	0.004^c^
	Post-test	51.09 (9.04)	53.44 (32–68)			41.05 (7.23)	39.59 (27–55)		10.04		0.001^c^
Urea level	Pre-test	112.58 (27.33)	114.00 (44–167)	−8.97	0.001^b^	106.29 (28.38)	114.00 (27–167)	17.82	−6.29	0.001^b^	0.287^d^
	Post-test	103.61 (19.79)	99.4 (54–158)			124.11 (34.31)	119.00 (45–217)		20.50		0.001^d^
Creatinine level	Pre-test	10.66 (3.37)	9.96 (3–17)	−0.87	0.006^b^	9.92 (3.16)	10.01 (3–17)	1.31	−0.74	0.001^b^	0.286^d^
	Post-test	9.78 (2.49)	9.58 (4–14)			11.23 (3.59)	12.10 (3–19)		1.44		0.030^d^

ᵃWilcoxon signed-rank test. ᵇPaired *t*-test. ᶜMann–Whitney U test. ᵈIndependent *t*-test, MD, Mean Difference.

As shown in Table 1, no significant differences were found between the intervention and control groups regarding baseline characteristics, including age, duration of hemodialysis, body weight, gender, marital status, education level, occupation, and comorbidities. These findings indicate that both groups were comparable and homogeneous prior to the intervention.

In Table 2, within-group comparisons using paired t-tests and Wilcoxon signed-rank tests showed that both groups experienced improvements in self-care scores. However, the intervention group demonstrated a significantly greater increase, with a mean score rising from 63.09 to 81.42 (mean difference = 18.33; *p* < 0.001), compared to the control group, which increased from 67.64 to 70.11 (mean difference = 2.47; *p* = 0.003). Between-group differences post-intervention, analyzed using the Mann–Whitney U test, were statistically significant (*p* = 0.001), with a large effect size (Cohen’s *d* = 0.932).

A similar pattern was observed for quality of life. The intervention group’s score increased significantly from 33.85 to 51.09 (mean difference = 17.25; *p* < 0.001), while the control group had a modest increase from 38.33 to 41.05 (mean difference = 2.73; *p* = 0.004). Between-group analysis revealed a significant difference (*p* = 0.001), with a large effect size (Cohen’s *d* = 1.226).

In terms of clinical outcomes, the control group’s urea levels increased from 106.29 to 124.11 (mean difference = +17.82; *p* < 0.001), while the intervention group’s levels significantly decreased from 112.58 to 103.61 (mean difference = −8.97; *p* < 0.001). Creatinine levels also decreased in the intervention group from 10.66 to 9.78 (mean difference = −0.87; *p* = 0.006), while the control group had an increase from 9.92 to 11.23 (mean difference = +1.31; *p* = 0.001). Independent t-tests revealed statistically significant between-group differences, and the corresponding effect sizes (Cohen’s *d* = 0.732 for urea, and 0.468 for creatinine) indicate moderate clinical impact.

## 4 Discussion

This study demonstrates that a structured health education program based on the PRECEDE–PROCEED model is associated with significant improvements in self-care behaviors, which constituted the primary outcome of the study, among patients undergoing hemodialysis (HD). Improvements in quality of life (QOL) and selected biochemical outcomes, including serum urea and creatinine levels, were observed as secondary and exploratory findings. These results contribute to the growing body of literature supporting theory-driven educational interventions in the management of chronic illnesses, particularly in resource-limited settings where patient empowerment and sustainable behavioral change are critical.

The PRECEDE-PROCEED model offers a comprehensive framework that begins with a diagnostic phase (PRECEDE) and culminates in the implementation and evaluation of interventions (PROCEED). Its strength lies in its systematic identification of factors that influence health behavior including predisposing, enabling, and reinforcing elements, which ensures that interventions are contextually relevant and individually responsive (23). In this study, the model was operationalized through targeted education across eight sessions, designed to foster knowledge, build self-efficacy, and support behavioral transformation. The intervention group experienced a notable increase in self-care scores (Cohen’s *d* = 0.932), reinforcing the robustness of the primary outcome and affirming the model’s capacity to facilitate behavioral adoption in populations with complex clinical and psychosocial needs, such as ESRD patients undergoing dialysis.

This result aligns with prior applications of the model in diverse chronic populations, including those with hypertension, type 2 diabetes, coronary heart disease, and postpartum conditions (24–26). What distinguishes the present study is its exploration of potential changes in selected physiological parameters alongside behavioral outcomes. However, biochemical outcomes were not predefined as primary endpoints and were assessed as exploratory indicators. Consequently, any observed changes in serum urea and creatinine should be interpreted cautiously and considered hypothesis-generating rather than confirmatory.

Improvements in quality of life (QOL), particularly across physical and mental health domains, were observed as secondary outcomes following the educational intervention. While QOL is an important patient-centered indicator in HD populations, these findings should be interpreted in relation to the primary behavioral improvements achieved through enhanced self-care and knowledge. The observed QOL changes may reflect downstream benefits of improved self-management capacity rather than direct effects of the educational program itself. Hemodialysis patients often experience profound impairments in social functioning, emotional well-being, and physical vitality due to the demanding nature of the therapy and its impact on daily life (27). By addressing knowledge gaps and psychological barriers while offering structured behavioral reinforcement, the intervention enhanced patient autonomy and self-management capabilities, leading to improvements that are both statistically and clinically meaningful (Cohen’s *d* = 1.226). This aligns with findings by Ngamdee et al. (28), which showed that behavioral interventions grounded in empowerment frameworks produce durable improvements in patient-perceived well-being.

The observed reductions in biochemical markers, specifically serum urea and creatinine levels, were noted following the educational intervention. However, these findings should be interpreted with caution. In patients undergoing hemodialysis, biochemical outcomes are influenced by multiple clinical factors beyond educational interventions, including dialysis adequacy, dialyzer membrane characteristics, ultrafiltration volume, and concurrent medication use. As these variables were not adjusted for in the present analysis, a direct causal relationship between the educational program and biochemical improvement cannot be established. Nevertheless, improved self-care behaviors and adherence to dietary, fluid, and pharmacological recommendations, as core targets of the PRECEDE-PROCEED-based educational program may plausibly contribute to more favorable biochemical profiles. This suggests that the intervention may have played an indirect or supportive role in the observed biochemical changes, rather than acting as an isolated determinant. Similar associations between behaviorally focused education and improved biochemical trends have been reported in previous studies (29). When considered alongside the substantial improvements observed in self-care behaviors and quality of life, these findings underscore the potential holistic value of structured educational frameworks in the comprehensive management of patients undergoing hemodialysis.

The observed effect sizes, particularly for self-care outcomes, suggest that structured, theory-based educational frameworks may offer advantages over less systematic educational approaches. However, given the exploratory nature of this study and the absence of adjustment for multiple comparisons, these findings should be interpreted as indicative rather than definitive. In resource-constrained health systems, where patient volumes are high and healthcare workforce capacity is limited, structured educational frameworks offer scalable solutions that can be adapted across patient populations (30). This culturally tailored intervention likely contributed to its success. Content was developed considering the cultural, linguistic, and literacy profiles of Indonesian HD patients. The repeated delivery during dialysis sessions optimized patient availability and reduced dropout rates. Moreover, the provision of standard booklets to both groups helped equalize baseline knowledge and avoided ethical concerns, while the differentiated content and structured reinforcement in the intervention group were instrumental in driving behavioral change (31, 32).

Despite its strengths, the study has several limitations that should be considered when interpreting the results. The intervention’s duration was relatively short, spanning only 4 weeks, which limits conclusions about the long-term sustainability of the improvements. Furthermore, there is a chance of bias when self-report measures are used for QOL and self-care. The study was also conducted in two hospital-based HD units in South Sulawesi, which could restrict the applicability of the results to broader, more diverse populations. To address these limitations, future research should incorporate longitudinal follow-up to assess the durability of behavior change, potentially integrating booster sessions or mobile health (mHealth) reinforcement to maintain gains over time. Furthermore, expanding the intervention to include family caregivers may offer added benefit, as social support is a known determinant of adherence and QOL in HD patients (33). Incorporating elements of telehealth or AI-based coaching platforms could also increase scalability and personalization of interventions in rural or underserved areas (34). Overall, this study provides preliminary evidence that a PRECEDE–PROCEED–based health education program may improve self-care behaviors as the primary outcome and is associated with secondary improvements in quality of life and selected biochemical parameters among patients undergoing hemodialysis. While the behavioral findings appear robust, the secondary and exploratory outcomes should be interpreted with caution due to the short intervention duration, absence of long-term follow-up, and lack of adjustment for multiple testing. Future studies with longitudinal designs and predefined clinical endpoints are warranted to confirm these findings. The structured, theory-driven approach appears to support targeted patient education and behavioral change within routine hemodialysis care settings. However, given the short duration of the intervention and the absence of long-term follow-up, the sustainability of the observed behavioral, quality-of-life, and biochemical changes cannot be established. Accordingly, these findings should be interpreted with caution, and their generalizability may be limited to similar clinical contexts.

Despite these limitations, the results suggest that structured, theory-based patient education has the potential to contribute meaningfully to comprehensive hemodialysis care. When appropriately integrated into standard care pathways, such educational approaches may support patient outcomes and could offer longer-term health system benefits, including the potential reduction of complications and healthcare utilization. Future research employing longitudinal designs, broader clinical settings, and adjustment for dialysis-related clinical variables is warranted to evaluate the long-term effectiveness and scalability of PRECEDE–PROCEED–based educational interventions in hemodialysis populations.

## Data Availability

The raw data supporting the conclusions of this article will be made available by the authors, without undue reservation.
